# Analysis of rituximab’s usage for the treatment of pediatric systemic lupus erythematosus

**DOI:** 10.1186/1546-0096-12-S1-P313

**Published:** 2014-09-17

**Authors:** Alessandra Alves, Ingrid HR Grein, Christina F Pelajo, Thais C Meneghetti, Loris L Janz Junior, Marcia Bandeira

**Affiliations:** 1 Reumatopediatria, Hospital Pequeno Príncipe, Curitiba, Brazil

## Introduction

Systemic lupus erythematosus (SLE) is a heterogeneous autoimmune disease characterized by chronic inflammation that potentially leads to organ damage. Severe manifestations make the management of SLE difficult and challenging. Nonsteroidal antiinflammatory drugs (NSAIDs), antimalarial drugs, glucocorticoids, immunosuppressive and citotoxic agents are the mainstays of treatment. Unfortunately, these agents are related to a vast toxicity. For an amount of patients, these approaches arent enough to achieve disease activity control, especially in those with cytopenia, nephritis and neuropsychiatric lupus. For these cases, Rituximab (RTX) – a chimeric mouse/human monoclonal antibody specific for human CD20 B-cell – has been widely prescribed. It is known that B-cells have a central role in SLE pathogenesis, being precursors of plasma cells production autoantibodies, precipitating inflammation by producing cytokines, activation T-cells by presentation of self-antigens, and regulating T-cells activity via co-stimulatory molecules. Although the clinical trials of RTX in SLE had controversial results, RTX seems to be a valuable option as an off-label drug for refractory patients.

## Objectives

To report the experience of a tertiary pediatric rheumatology center with RTX for patients with refractory SLE.

## Methods

We performed a retrospective chart review of patients with SLE that received RTX in some moment of their disease from September 2009 to February 2013 due to refractory to traditional therapies. A satisfactory response was defined as reaching SLEDAI reduction in at least 50%, urina protein:creatinine ratio (UPCR) <50 mg/mmol (equivalent to proteinuria <0.5g/24h), and corticoid tapering to at least 50% of inicial dose.

## Results

Seven patients were in use of the medication in the revised period. All of then were female. Six patients had the diagnosis of lupus nephritis, and 1 had refractory plaquetopenia to standart drugs. Two patients had an inadequate response to RTX. One of them persisted with refractory plaquetopenia and the other died due to complications related to SLE. Out of the 6 patients with lupus nephritis, 4 (66%) achieved SLEDAI reduction of at least 50% after 4-6 months of RTX, as well showed an important reduction of proteinuria (<0.5g/24h) and corticoid tapering. One patient had improvement of her disease, however without achieving the paramethers of satisfactory response to the RTX. For this reason, this patient was considered to have a partial response to the RTX. The results are according to those found in similar studies.

## Conclusion

It is suggested that RTX continues to be used as a therapeutic option for patients with refractory SLE or with contraindication to traditional drugs. Prospected studies are still needed to evaluate efficacy and safety of RTX in pediatric patients.

## Disclosure of interest

None declared.

